# Immunogenicity, Protective Efficacy, and Non-Replicative Status of the HSV-2 Vaccine Candidate HSV529 in Mice and Guinea Pigs

**DOI:** 10.1371/journal.pone.0121518

**Published:** 2015-04-02

**Authors:** Marie-Clotilde Bernard, Véronique Barban, Fabrine Pradezynski, Aymeric de Montfort, Robert Ryall, Catherine Caillet, Patricia Londono-Hayes

**Affiliations:** 1 Sanofi Pasteur, Research and Development, Marcy-l’Étoile, France; 2 Sanofi Pasteur, Research and Development, Swiftwater, Pennsylvania, United States of America; UC Irvine Medical Center, UNITED STATES

## Abstract

HSV-2 vaccine is needed to prevent genital disease, latent infection, and virus transmission. A replication-deficient mutant virus (*dl5-29*) has demonstrated promising efficacy in animal models of genital herpes. However, the immunogenicity, protective efficacy, and non-replicative status of the highly purified clinical vaccine candidate (HSV529) derived from *dl5-29* have not been evaluated. Humoral and cellular immune responses were measured in mice and guinea pigs immunized with HSV529. Protection against acute and recurrent genital herpes, mortality, latent infection, and viral shedding after vaginal HSV-2 infection was determined in mice or in naïve and HSV-1 seropositive guinea pigs. HSV529 replication and pathogenicity were investigated in three sensitive models of virus replication: severe combined immunodeficient (SCID/Beige) mice inoculated by the intramuscular route, suckling mice inoculated by the intracranial route, and vaginally-inoculated guinea pigs. HSV529 immunization induced HSV-2-neutralizing antibody production in mice and guinea pigs. In mice, it induced production of specific HSV-2 antibodies and splenocytes secreting IFNγ or IL-5. Immunization effectively prevented HSV-2 infection in all three animal models by reducing mortality, acute genital disease severity and frequency, and viral shedding. It also reduced ganglionic viral latency and recurrent disease in naïve and HSV-1 seropositive guinea pigs. HSV529 replication/propagation was not detected in the muscles of SCID/Beige mice, in the brains of suckling mice, or in vaginal secretions of inoculated guinea pigs. These results confirm the non-replicative status, as well as its immunogenicity and efficacy in mice and guinea pigs, including HSV-1 seropositive guinea pigs. In mice, HSV529 produced Th1/Th2 characteristic immune response thought to be necessary for an effective vaccine. These results further support the clinical investigation of HSV529 in human subjects as a prophylactic vaccine.

## Introduction

Genital herpes is an important global health problem usually caused by a sexually transmitted infection with herpes simplex virus type 2 (HSV-2), although infection by type 1 (HSV-1) is becoming increasingly common [1]. Globally, over 536 million people were estimated to be living with HSV-2 infection in 2003, with over 23 million new infections [2]. HSV-2 infection is a lifelong disease because the latent virus persists in neural ganglia. It frequently manifests as episodic outbreaks of painful genital lesions caused by viral reactivation, which is also when individuals are most infectious. Although the great majority of infections are asymptomatic, seropositive individuals can also shed virus and represent a large pool of transmissible virus [1]. The risk of acquiring human immunodeficiency virus (HIV) is higher by 2−3-fold higher in HSV-2-seropositive persons [3, 4]. Pregnant women with an active HSV-2 infection can transmit the virus during delivery to their neonate which can result in severe neurological disease or neonatal death. Seroprevalence is up to twice as high in women as in men and 2003 regional estimates for HSV-2 infection in women were 17.9% in North America and 13.7% in Western Europe, with highs of 61.8% in East Asia and 78.2% in Sub-Saharan Africa [2].

Treatment options for HSV-2 infection are essentially limited to the antiviral drug acyclovir, which provides measurable symptomatic relief and reduces transmission. Prophylactic acyclovir treatment also reduces asymptomatic virus shedding and transmission but does not eliminate them [5]. Thus, an HSV-2 vaccine is needed to prevent herpes disease and the spread of the virus. A number of HSV-2 glycoprotein-based vaccine candidates have shown promise in animal models but have not been consistently effective in clinical trials. In two early trials, a recombinant HSV-2 glycoprotein D vaccine in an aluminum hydroxide and 3-O-deacylated monophosphoryl lipid adjuvant was 73% and 74% efficacious only in women seronegative for both HSV-1 and HSV-2 but ineffective in HSV-1-seropositive women and in men [6]. More recently, the results of a large phase III trial of this vaccine conducted in over 8300 HSV-1- and HSV-2- seronegative women were disappointing, as the vaccine was 58% efficacious against genital disease caused by HSV-1 was but was not efficacious against HSV-2 disease [7]. These data suggest that different kinds of vaccine candidate are needed. Even if the profile of protective immune response is still not fully understood, a vaccine that mimics natural viral infection and induces broad humoral and CD4+ and CD8+ T cell responses would likely be a good vaccine candidate.

In mouse and guinea pig models of genital herpes, a live attenuated, replication-deficient HSV-2 variant, *dl5-29*, induces humoral and cellular immune responses and protects against HSV-2 infection, genital lesions, virus shedding, recurrence, and viral latency [8]. A purified GMP version of this vaccine candidate, ACAM529, has demonstrated similar immunogenicity, protective efficacy, and undetectable replication in mice and guinea pigs [9].

The vaccine preparation to be used for clinical investigation (HSV529) is a plaque-purified clone of the ACAM529 virus produced at large scale and highly purified under GMP conditions. This study was performed to confirm the immunogenicity, protective efficacy, and lack of replication of the HSV529 clinical vaccine candidate in mice and guinea pigs prior to entering phase I clinical evaluation.

## Materials and Methods

### Viruses and cell lines

Construction of the original replication-deficient HSV-2 *dl5-29* virus from the HSV-2 186 syn^+^-1 parent strain has been described [10]. The virus lacks two viral DNA replication genes (*U*_*L*_ *5* and *U*_*L*_ *29*) and is completely defective in DNA synthesis and viral replication in the absence of these two gene products. The virus is grown on the complementary helper cell line AV529-19 derived from the Vero cell line (ATCC, CCL-81.2), which was stably transfected to supply the UL5 and UL29 HSV-2 proteins *in trans* in infected cells [11]. Production of highly purified ACAM529 virus from a *dl5-29* clone has been described [9]. To produce HSV529 vaccine, an ACAM529 clone underwent four additional rounds of plaque purification and a single clone was produced at large scale and purified under GMP conditions. The AV529-19 cell line was maintained in Dulbecco’s Modified Eagle Medium F12 (Life Technologies, Carlsbad, CA) supplemented with 4 mM glutamine, 0.5X Cholesterol Lipid Concentrate (Life Technologies, Carlsbad, CA), 0.5 mg/mL G418, 50 mM sucrose, and 10% fetal calf serum in a 5% CO_2_ atmosphere. Wild-type HSV-2 strains (G, 333, and 186 syn^+^-1) and the wild-type HSV-1 strain (KOS) were grown on Vero cells that were maintained in Dulbecco’s Modified Eagle Medium supplemented with 10% fetal calf serum in a 5% CO_2_ atmosphere.

### Virus titration

A viral plaque assay was used to titrate wild-type HSV strains on Vero cells and the HSV529 vaccine candidate on AV529-19 cells. Virus preparations were titrated before and after inoculation by serially diluting the sample tenfold and plating the dilutions in 96-well plates seeded with the cell line appropriate for the viral strain. After 2−3 days of culture, infected cells were fixed with methanol and immunostained with a HSV glycoprotein D antibody (Virusys Corporation) and anti-mouse IgG-alkaline phosphatase antibody conjugate (Southern Biotech), and developed with Sigmafast BCIP/NBT (Sigma-Aldrich, Saint Louis, MO) chromogenic reagents. The viral titer was determined from the dilution that infected 50% of the cells in the well (CCID_50_) and expressed as the number of CCID_50_ units in the solution or sample.

### Ethical use of animals

All animal experiments were performed in compliance with European Directive 2010/63 and national regulations. Studies on BALB/c ByJ mice were conducted in animal facilities accredited by the Association for Assessment and Accreditation of Laboratory Animal Care International. The protocols were approved by the Committee on the Ethics of Animal Experiments of the Sanofi Pasteur France or Institut Bourgelat and all efforts were made to reduce the use of animals and to minimize pain and distress.

### Mouse immunizations and herpes model

Nine-week-old female BALB/c ByJ mice (Charles River Laboratories, Saint Germain-sur-l’Arbresle, France) received two intramuscular (i.m.) injections (50 μL), three weeks apart, of either phosphate-buffered saline (PBS) or HSV529 in the quadriceps muscle. HSV529 doses between 10^4^ and 10^6^ CCID_50_ were evaluated for immunogenicity and a dose of 10^6^ CCID_50_ was used to evaluate protection. Blood samples and spleens were collected 1 or 3 weeks after the second injection of HSV529 to evaluate cellular and humoral immune responses.

In protection assays, mice received two subcutaneous (s.c.) injections of medroxyprogesterone acetate (2 mg in 100 μl; Depo-Provera, Pfizer) seven days and one day before HSV-2 vaginal challenge to block their reproductive cycle in diestrus. Anesthetized mice were inoculated in the vaginal vault with HSV-2 G strain (10^5^ CCID_50_; 50 LD_50_) in a volume of 20 μL at week 7.

### Guinea pig immunizations and herpes model

Four- to six-week-old female Dunkin Hartley guinea pigs (Charles River Laboratories, Saint Germain-sur-l’Arbresle, France) were used to evaluate HSV529 immunogenicity and protection in the HSV-2 vaginal challenge model. HSV-1-naïve guinea pigs received two intramuscular (i.m.) injections (100 μL), three weeks apart, of either phosphate-buffered saline (PBS) or 10^6^ CCID_50_ of HSV529 vaccine in the quadriceps muscle. For the HSV-2 challenge on day 48, the animals were anesthetized and the vaginal vault was cleaned with a swab. Wild-type HSV-2 G strain (100 μL containing 10^5^ CCID_50_) was slowly deposited in the vaginal vault. Animals were monitored daily for disease severity and on days 1−4, 7, and 10 for viral shedding by titration of the vaginal secretions collected on cotton swabs. Genital lesions arising after day 14 were considered to be recurrent and were monitored through post-infection (p.i.) day 65 or 68. At the end of the experiment, lumbosacral ganglia were assessed for latent HSV-2 DNA by quantitative PCR (qPCR). Blood samples were collected throughout each experiment for immunogenicity analyses.

To evaluate protection in HSV-1-seropositive animals, 4−6-week-old female Dunkin Hartley guinea pigs were first intranasally inoculated with HSV-1 KOS strain (10^6^ CCID_50_) or PBS on day 0 and then evaluated for HSV-1 seroconversion by HSV-2 specific IgG ELISA at week 4. Seropositive animals were randomized into two groups of 15 animals with equivalent geometric mean antibody titers and immunized i.m. with either PBS (100 μl) or HSV529 vaccine (10^6^ CCID_50_) at weeks 7 and 10. At week 14, to ensure genital disease in these animals, they were challenged with a vaginal dose of wild-type HSV-2 (2 x 10^6^ CCID_50_) 20-fold higher than the dose used for naïve guinea pigs [12]. After challenge, animals were monitored as in the naïve guinea pig model. Blood samples were collected throughout each experiment for immunogenicity analyses.

### Herpes disease severity

#### Acute phase

On observation days, the scoring of herpetic lesions in mice and guinea pigs was evaluated using a 5-point scale: from 0 for no sign of disease to 5 for severe localized herpetic lesions. Humane end-points were used to prevent unnecessary suffering of the animals. Animals exhibiting 20% loss in body weight, hind leg paralysis, severe neurological signs of urinary or fecal retention, or profound distress were euthanized.

#### Latent phase

The recurrent disease phase in guinea pigs started as soon as p.i. day 14 or 15 before they had completely recovered from the acute phase. Animals were observed on weekdays through p.i. day 65 or 68. Recurrent lesions were mild and never exceeded one single vesicle or pustule or a score of 2 or 3 on the lesion scale.

### IgG1 and IgG2a ELISA

96-well microplates (Dynex Technologies, West Sussex, UK) were coated overnight at 4°C with 200 ng/well (100 μL) of HSV-2 G strain Purified Viral Lysate (Advanced Biotechnologies, Columbia, MD) in 0.05 M sodium carbonate buffer pH 9.6. Plates were then blocked for 1 hour at 37°C with 150 μL of phosphate buffered saline (PBS) pH 7.1 containing 0.05% Tween 20 and 1% (w/v) powdered skim milk (PBS-Tween-milk). All further incubations were carried out in a final volume of 100 μL, followed by 4 washings with PBS, pH 7.1 containing 0.05% Tween 20. Serum samples that were serially diluted twofold in PBS-Tween-milk beginning at 1/100 or 1/1000 were added to the wells and incubated for 90 min at 37°C. After washing, HRP-conjugated antibodies to mouse IgG1 (Southern Biotech, Birmingham, AL) or IgG2a (Southern Biotech,) were added and the plates were incubated for another 90 min at 37°C followed by washing and color development with TMB substrate (Tebu-Bio Laboratories, Le-Perray-en-Yvelines, France). The optical density (OD) was measured at 450–650 nm with an automatic plate reader (Molecular Devices, Sunnyvale, CA). Antibody titers were calculated using the CodUnit software (Dipole, France) for OD values of 0.2−3.0, from a standard curve established with anti-HSV mouse reference serum. The titer of this reference had been previously determined after several experiments and is expressed as the average of the reciprocal dilution which gives an OD of 1.0 in each assay. The threshold of antibody detection was 20 (1.3 log_10_) ELISA units (EU). All final titers were expressed in log_10_. Titers < 1.3 log_10_ were assigned a value of 1.0 log_10_.

### Seroneutralization assay

HSV-2-neutralizing antibodies in the sera of immunized animals were titrated by mixing dilutions of heat-inactivated sera (56°C for 30 min) with an equal volume of medium containing HSV-2 (G strain, approximately 100 CCID_50_/well) and 10% baby rabbit complement, and then incubating for 1 hour at 37°C. Polyclonal rabbit anti-HSV antiserum (Acris Antibodies GmbH, Herford, Germany) was used as a positive control in the guinea pig assay and naïve sera were included in each assay. An aliquot of serum/virus mixture (50 or 100 μL) was then added to Vero cell monolayers in flat bottom 96-well plates and incubated in a 37°C, 5% CO_2_ cell culture incubator for 20 h for the guinea pig assay or for 2−3 days for the mouse assay. Infected cells were fixed with methanol and immunostained with anti-HSV glycoprotein D antibody (Virusys Corporation, Taneytown, MD), anti-mouse IgG alkaline phosphatase (Southern Biotech), and developed with BCIP/NBT (Sigma-Aldrich) chromogenic reagents. The images of the wells were acquired using an ELISPOT reader (Microvision Instruments, France). Wells containing plaques were detected with Celest software (Microvision Instruments, France) for the mouse assay and individual plaques in each well were counted with Spot software (Microvision Instruments, France) for the guinea pig assay. Titers were calculated from the dilution that resulted in 50% neutralization (SN_50_) by using a least-square regression method (mouse assay) or a 4-parameter method (guinea pig assay) and were expressed as the reciprocal of the log_10_-transformed value. Guinea pig SN_50_ geometric mean titers were determined for each group from serum dilutions tested once in a single assay. Mouse SN_50_ values were obtained from serum dilutions tested in quadruplicate and in two independent assays. The LLOD was of 1.0 log_10_ SN_50_ titer and values below this were assigned a value of 0.7 log_10._

### Quantification of HSV-2-reactive lymphocytes secreting IFNγ or IL-5

96-well nitrocellulose-bottomed microplates (Millipore, Billerica, MA) were coated with a rat antibody specific for mouse IFNγ or for mouse IL-5 (BD Pharmigen, Le Pont de Claix, France). After plate washing, freshly isolated splenocytes (2 x 10^5^ cells/well) from immunized and control BALB/c ByJ mice were added to the plate and incubated overnight with murine IL-2 (10 μg/mL; Roche-Boehringer Mannheim, Meylan, France) and either heat-inactivated HSV-2 strain G (10^5^ CCID_50_/mL equivalents), recombinant HSV gD2 (1 μg/mL; Mybiosource, San Diego, CA), concanavalin A (2.5 μg/mL) as a positive control, or medium as a negative control. The plates were washed and the locations where cells secreting INFγ or IL-5 during incubation were stained with a biotinylated anti-mouse IFNγ or IL-5 antibody (BD Pharmigen, Le Pont de Claix, France), a streptavidin-horseradish peroxidase conjugate (Southern Biotechnology), and a chromogenic substrate (amino ethyl carbazole; Sigma-Aldrich, St. Louis, MO). Plates were read with an automatic ELISPOT reader (Microvision Instruments, France) and stained cells were counted with Spot software (Microvision Instruments, France). Results were expressed as the number of cells (spots) per 10^6^ splenocytes. The threshold of positivity was established at 20 cells/10^6^ splenocytes.

### Viral shedding

Samples were collected by inserting a swab (CleanTips Swab, Micro CleanFoam Head, ITW Texwipe, VWR Cat. No. TWTX757B for mice; sterile swab applicator, Copan 167KS01 086A19 for guinea pigs) that had been pre-wet with swab buffer (PBS containing 0.1% glucose, 1% heat-inactivated fetal calf serum, 1 U/ml penicillin, and 1 μg/ml streptomycin) into the vaginal vault, vortexing the swab in swab buffer (0.5 ml for mice or 1.0 ml for guinea pigs), and storing at −80°C until virus titration assay by the CCID50 log_10_ method. Shed virus measured at p.i. day 1 was considered to reflect a combination of residual virus from the inoculum and newly formed virus from the beginning of a productive infection. Shedding between p.i. days 2 and 3 was considered to be newly formed virus and to indicate the extent of mucosal infection. The positive threshold value was of 2.5 log_10_ CCID_50_/mL. Samples with titers below the positive threshold were assigned a value of 2.2 log_10_ CCID_50_/mL.

### Intramuscular HSV529 replication and/or propagation in SCID/Beige mice

Six-week-old female Fox Chase SCID Beige mice (CB17.Cg-Prkdc^scid^ Lyst^bg^; Charles River Laboratories, Saint Germain-sur-l’Arbresle, France) were used to test for HSV529 propagation in mice with severe B, T, and natural killer cell immunodeficiency and were housed in individually ventilated ISOcages (Tecniplast, France). Mice were inoculated with an i.m. injection (50 μL) of PBS, heat-inactivated HSV529 (4 x 10^6^ CCID_50_ equivalents), HSV529 (4 x 10^6^ CCID_50_), or HSV-2 186 syn^+^-1 (500 CCID_50_). Animals were observed daily for paralysis, neuronal symptoms or death, but handling was reduced to minimum. Gastrocnemius muscles were collected from some animals 4 hours after inoculation, from animals that died or were euthanized prior to day 28, and from surviving animals on day 28. Muscles were homogenized using a Precellys24 bead-based homogenizer and the MK28 Lysis Kit (Bertin Technologies, France). To detect infectious viral particles, 40% of each muscle sample homogenate was incubated for 2 hours on AV529-19 cell monolayers in a 37°C tissue culture incubator. Cells were washed once with cell culture medium and cultured for 6 days. If no CPE was observed after the first passage, cells were lysed with three freeze-thaw cycles plus sonication, and 50% of the crude lysate was cultured on a fresh AV529-19 cell monolayer for 6 days. If necessary, amplification was repeated once more for a total of three passages. For samples with detectable CPE after three passages on AV529-19 cells, the virus in 50% of the culture lysate and in 40% of the original muscle homogenate was tested for replication competency by three successive passages on Vero cells. Samples were considered negative for propagating virus if CPE was undetectable after three passages. The lower limit of detection (LLOD) in muscle homogenate was determined empirically and estimated to be 40 CCID_50_ for the HSV529 vaccine on AV529-19 cells and 70 CCID_50_ for HSV-2 186 syn+-1 on AV529 19 cells or Vero cells.

### Cranial HSV529 replication in suckling mice

Four- to six-day-old suckling mice (BALB/c ByJ, haplotype H2-Db, specific pathogen-free, Charles River Laboratories, Saint Germain-sur-l’Arbresle, France) were anesthetized by exposure to 4.5% isoflurane for 2 minutes prior receiving an intracranial injection (20 μL) of vaccine buffer (10% sucrose, 50 mM potassium glutamate, 160 mM sodium chloride, 10 mM histidine, pH 7.0), HSV529 (5 x 10^5^ CCID_50_), or HSV-2 186 syn+-1 (10 CCID_50_). Animals were monitored daily and brains were collected from animals and from euthanized animals on days 0 (4 h after injection), 2, 4, 6, and 14. Brains were placed in cryotubes containing 500 μL of PBS containing 0.1% glucose and 1% heat-inactivated FCS, immediately frozen in dry ice, and stored at −80°C until viral titrations. Brains from were homogenized in Precellys 24 bead-based homogenizer using CKMix Lysis Kit (Bertin Technologies, France). The viral titers per brain were determined by CCID_50_ titration on AV529-19 cells. The LLOD in brain homogenate was determined empirically and estimated to be 3.4 log_10_ CCID_50_/brain. Samples with titers below the LLOD were assigned a value of 3.1 log_10_ CCID_50_/brain.

### Vaginal HSV529 replication and/or propagation in guinea pigs

Four- to six-week-old female Dunkin Hartley guinea pigs (specific pathogen-free; Charles River Laboratories, Saint Germain-sur-l’Arbresle, France) were vaginally inoculated with PBS (100 μL), heat-inactivated HSV529 (2 x 10^7^ CCID_50_ equivalents), HSV529 (2 x 10^7^ CCID_50_) or HSV-2 186 syn+-1 (6300 CCID_50_) as described for guinea pig vaginal herpes model. On p.i. days 0, 3, 5, 7, 10, 13, and 31, vaginal secretions were collected as for the viral shedding assay. To detect infectious particles in vaginal secretions, 20% of each swab sample was seeded on Vero and on AV529-19 cell monolayers and incubated for 2 hours in a cell culture incubator. Cells were washed once and incubated for 6 days. If no CPE was observed on either cell type, Vero cells were lysed by three freeze-thaw cycles and sonication, and 50% of the lysate was seeded onto fresh Vero cell monolayers. Two rounds of amplification on Vero cells were performed before concluding that the sample contained no propagating virus. Amplifications were not done on AV529-19 cells. The LLOD was determined empirically and estimated to be ≤ 10 CCID_50_/mL on AV529-19 cells and ≤ 40 CCID_50_/mL on Vero cells.

### Quantitation of viral DNA in lumbosacral dorsal root ganglia

Lumbosacral ganglia from HSV-2 infected guinea pigs were homogenized with a Precellys 24 bead-based homogenizer using CKMix Lysis Kit (Bertin Technologies, France). Total genomic DNA was purified with NucleoSpin Tissue kit (Macherey-Nagel, Düren, Germany) according to the manufacturer’s protocol. Latent viral DNA was quantified by real-time quantitative PCR using Taqman technology, a Mx3005P qPCR instrument (Agilent Technologies, La Jolla, CA), HSV-2 gD2 primers: forward primer 5' GGA GAC AAT TGC GCT ATC 3’, reverse primer 5' CAG GAA TCC CAG GTT ATC 3’ (Eurofins MWG Operon, Ebersberg, Germany), and a gD2 Taqman MGB probe: 5’ FAM - CAC GGT TAT GGA ATA CAC - MGB 3'(Applied Biosystems—Life Technologies, Grand Island, NY). The LLOD was approximately 24 viral DNA copies per reaction, which extrapolated to 480 copies/animal. Samples with results below the LLOD were assigned a value of 240 copies/animal.

### Statistical analyses

All analyzes were performed using SAS v9.2 software (Cary, NC). Statistical analyses of comparisons between groups were based on the alternative hypothesis that the means, medians or proportions between groups were different. All the tests were performed at alpha risks of 5% for the main effects and 10% for the interaction effects. P-values lower than these values were considered to indicate statistically significant differences. Acute vaginal lesion scores were analyzed by calculating the area under the curve of the mean lesion score vs days after challenge for each animal during the entire acute phase (day 0−15) and comparing the values using a one-way analysis of variance model. Post-challenge titers of shed virus were compared in mice using an analysis of variance for each time point and compared in guinea pigs using an analysis of covariance model for repeated measures over time, with treatment groups as categorical variables and time as a continuous variable. For experiments in which shed virus was not detected in some animals, a non-parametric Wilcoxon test was used to compare the viral titers measured at each time point. Cumulative numbers of recurrent lesions for treatment groups were compared using an analysis of covariance with repeated measures over time, with the treatment groups as categorical variables and post-challenge time as a continuous variable. The percentages of animals in each group with at least one recurrence were compared using Fisher’s exact test. The percentages of animals positive for HSV-2 DNA in lumbosacral ganglia were compared using a Chi-square test. HSV-2 DNA totals (in log_10_ copies/animal) were compared using a one-way analysis of variance model. Body weight changes following HSV-2 challenge were compared using an analysis of variance model for repeated measures over time, with the treatment group as a categorical variable and post-challenge time as a continuous variable. Titers for IgG1 and IgG2a ELISA and seroneutralization assays were compared using an analysis of covariance model for repeated measures over time with the treatment group as a categorical variable and post-challenge time as a continuous variable.

## Results

**Fig 1:**
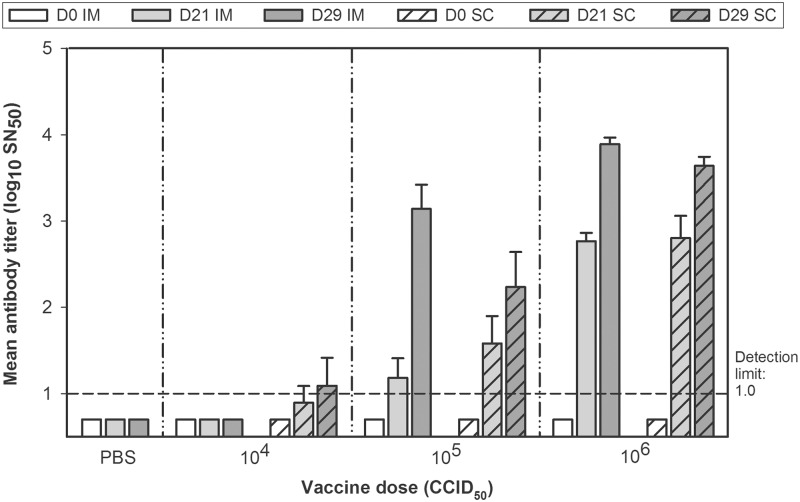
Guinea pigs immunized with HSV529 produce antibodies that neutralize HSV-2 infection *in vitro*. Guinea pigs were immunized with HSV529 (10^4^ CCID_50_, 10^5^ CCID_50_, or 10^6^ CCID_50_) by the intramuscular (IM; n = 5 each) or subcutaneous (SC; n = 5 each) route or with PBS (n = 3) by the intramuscular route on days 0 (D0) and 21 (D21). Sera were collected from all animals on days 21 and 29 (D29) and measured for HSV-2 neutralizing activity by preincubating dilutions of heat-inactivated sera with 100 CCID_50_ of live HSV-2 virus for 1 hour prior to infection of Vero cell cultures. Infected cells were detected with anti-HSV glycoprotein D antibodies. The serum dilution that neutralized 50% of the virus (SN50) was determined by plotting the neutralization activity versus the serum dilutions. Error bars represent standard error of the mean.

HSV529 is capable of generating strong cellular and humoral immune responses to HSV-2 virus. In all previous studies of *dl5-29* immunogenicity in guinea pigs, immunization was performed by the subcutaneous (s.c.) route [8, 12, 13]. However, the HSV529 vaccine candidate is being developed for delivery by the i.m. route, which has been effective in mice [11]. We therefore investigated the seroneutralizing immunogenicity of HSV529 (10^4^−10^6^ CCID_50_) delivered by both routes in guinea pigs (Fig. 1). Although the 10^4^ CCID_50_ dose did not induce a seroneutralizing antibody response, 21 days after being immunized once with 10^5^ or 10^6^ CCID_50_ by either delivery route, guinea pigs produced similar dose-dependent titers of antibodies capable of neutralizing wild-type HSV-2 virus (G) *in vitro*. Eight days after administration of a second dose, seroneutralization titers increased further but the magnitude of these increases varied according to dose and route of administration. Globally, after the second immunization, seroneutralization responses were significantly greater with the i.m. than with the s.c. route by an overall mean of 4-fold (p = 0.02, analysis of covariance).

**Fig 2:**
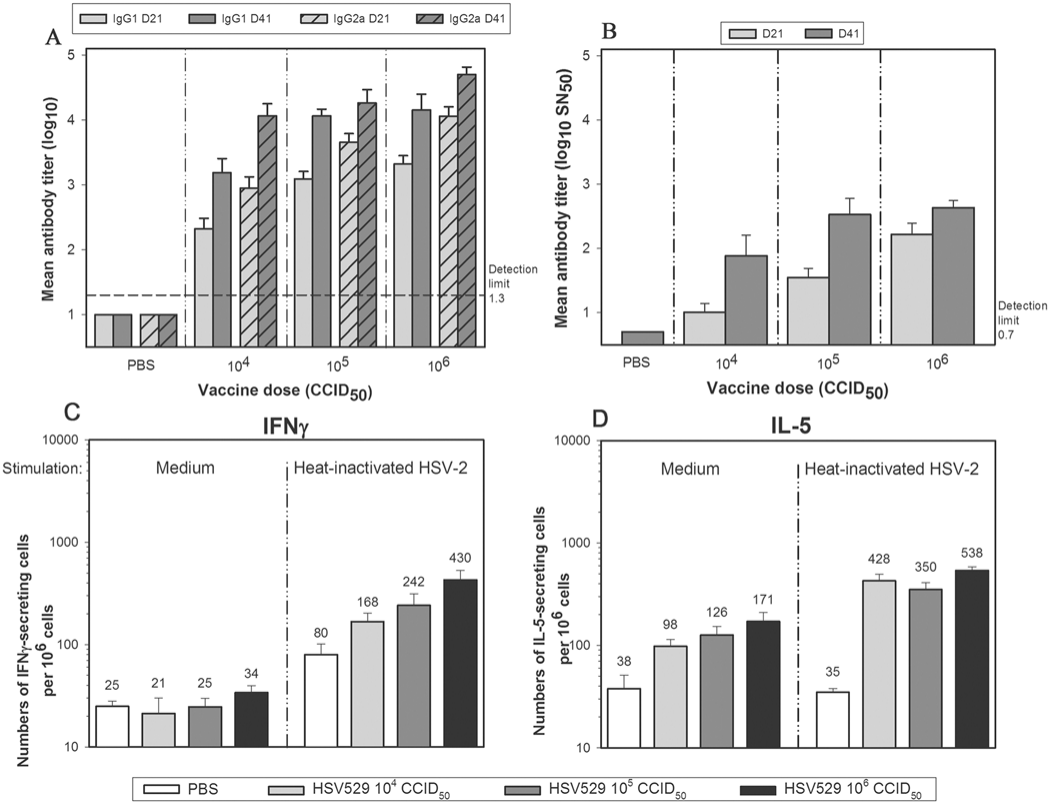
Mice immunized with HSV529 produce HSV-2-specific IgG1 and IgG2a antibodies, neutralizing antibodies, and HSV-2-specific splenic lymphocytes secreting IFNγ and IL-5. BALB/c mice (n = 10/group) were immunized with HSV529 (10^4^ CCID_50_, 10^5^ CCID_50_, or 10^6^ CCID_50_) or PBS by the i.m. route on days 0 and 21. Sera were collected on days 21 (D21; n = 10) and 41 (D41; n = 5). (A) HSV-2-specific IgG1 and IgG2a antibody titers in the sera were determined by ELISA using a lysate prepared from HSV-2-infected Vero cells and secondary antibodies specific for mouse IgG1 and IgG2a. (B) HSV-2 neutralizing antibodies in the sera were measured by preincubating dilutions of heat-inactivated sera with 100 CCID_50_ of live HSV-2 (strain G) virus for 1 hour prior to infection of Vero cell cultures. Infected cells were detected with anti-HSV glycoprotein D antibodies. The serum dilution that neutralized 50% of the virus (SN_50_) was determined by plotting the neutralization activity versus the serum dilutions. Splenic lymphocytes secreting IFNγ (C) or IL-5 (D) in response to *ex vivo* stimulation with heat-inactivated HSV-2 (strain G) were counted using an ELISPOT assay. Error bars represent standard error of the mean.

We next investigated the details of the immune response to HSV529 immunization in mice. Mice immunized with HSV529 (10^4^−10^6^ CCID_50_) by the i.m. route exhibited a dose-dependent immune response. The immune response includes both Th1 and Th2 characteristics (Fig. 2A).

At day 21, HSV-2-specific ELISA titers were between 2.9 log_10_ EU and 4.1 log_10_ EU for IgG2a and between 2.3 log_10_ EU to 3.3 log_10_ EU for IgG1. Twenty days after a second immunization at day 21 (i.e. at day 41), anti-HSV-2 ELISA titers for both antibody subtypes increased to between 4.1 log_10_ EU and 4.7 log_10_ EU for IgG2a and to between 3.2 log_10_ EU and 4.2 log_10_ EU for IgG1. HSV529 immunization in mice also induced dose-dependent production of HSV-2 seroneutralizing antibodies, which were evident after 21 days and also boosted by a second immunization (Fig. 2B). Immunization also produced strong Th1 and Th2 cellular immune response characterized by elevated frequencies of splenocytes that secreted either IFNγ or IL-5 in response to *ex vivo* stimulation with heat-inactivated HSV-2 virus (Fig. 2C and 2D). Whereas the production of IFNγ-secreting cells was dose-dependent, the production of IL-5-secreting cells was near maximal at the lowest dose of HSV529 (10^4^ CCID_50_). These results indicate that HSV529 immunization generated strong Th1 and Th2 humoral responses capable of neutralizing HSV-2 *in vitro* and T-cell-mediated helper responses characterized by HSV-2-specific lymphocytes capable of secreting IFNγ and IL-5 in response to the virus.

### HSV529 immunization protects BALB/c ByJ mice against HSV-2 infection, genital lesions, viral shedding and lethality

**Fig 3:**
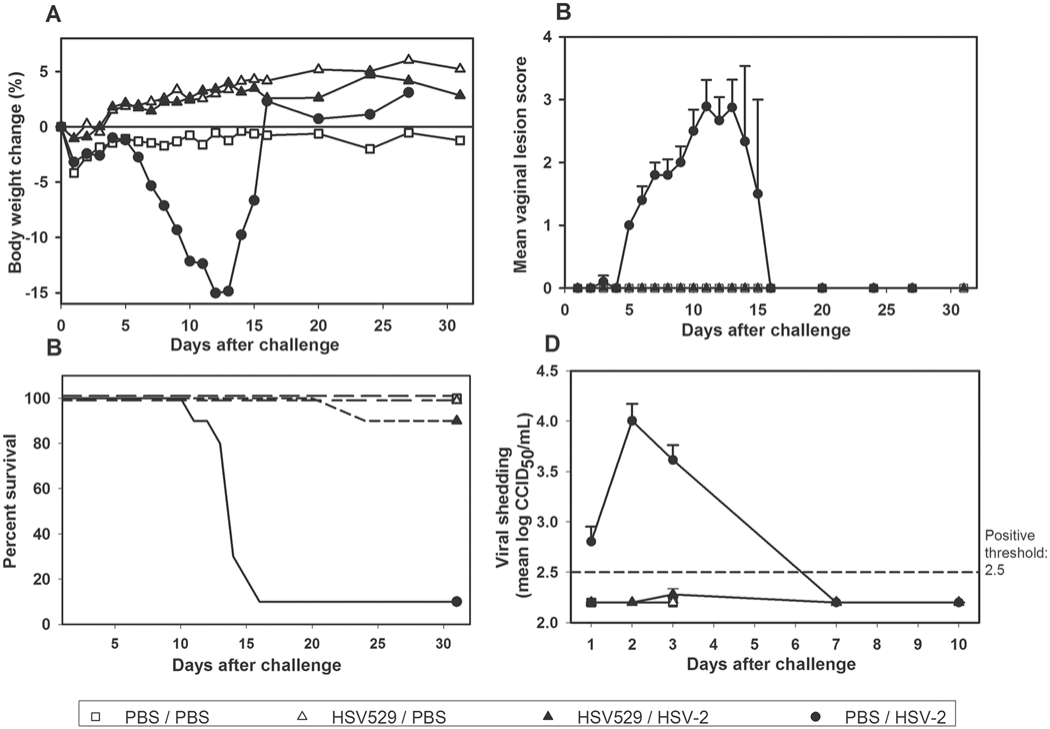
HSV529 immunization protects mice from the effects of lethal HSV-2 vaginal challenge. BALB/c mice were immunized with HSV529 (10^6^ CCID_50_) or PBS by the i.m. route on days 0 and 21. On day 48, mice received medroxyprogesterone (2 mg, s.c.) to prevent reproductive cycling. The next day, mice were challenged with an intravaginal inoculation of HSV-2 (strain G; 10^5^ CCID_50_). (A) Mean body weight change after HSV-2 challenge. (B) Mean vaginal lesion score after HSV-2 challenge. (C) Percent survival after HSV-2 challenge. (D) HSV-2 viral shedding after challenge. *Dead or euthanized animal. Error bars represent the standard error of the mean.

Vaginal HSV-2 infection in mice results in acute disease and a high rate of lethality [13]. Infected animals also lose body weight and shed virus. Following vaginal challenge with a dose of HSV-2 (G strain) equivalent to 50 LD_50_ (10^5^ CCID_50_), non-immunized control animals lost significant mean body weight between p.i. days 6 and 27 (p < 0.05, analysis of variance) with a maximum loss of up to 15% on p.i. day 12 (Fig. 3A). They also developed maximum mean lesion scores of nearly 3 between p.i. days 11 and 13 (Fig. 3B), and 90% of them (n = 10) died or were euthanized between p.i. days 11 and 16 (Fig. 3C). In contrast, two immunizations with HSV529 (10^6^ CCID_50_, i.m.) before HSV-2 vaginal challenge completely protected mice from subsequent weight loss (n = 10) and vaginal lesions (n = 10), and protected 90% of them from lethality (n = 10). The mean titer of shed virus on p.i. day 2 was 4.0 ± 0.7 log_10_ CCID_50_/mL in control animals, of which 19 out of 20 (95%) shed virus above the LLOD (Fig. 3D). In HSV529-immunized animals, the mean titer of shed virus remained below the positive threshold value of 2.5 log_10_ CCID_50_/mL through p.i. day 10 and was significantly lower than in the control group (p < 0.001, variance analysis). Fewer animals in this group (2 out of 20; 10%) shed virus above the LLOD and only on p.i. day 3.

### HSV529 immunization protects naïve guinea pigs against HSV-2 infection, genital lesions, viral shedding, disease recurrence, mortality, and latent infection

**Fig 4:**
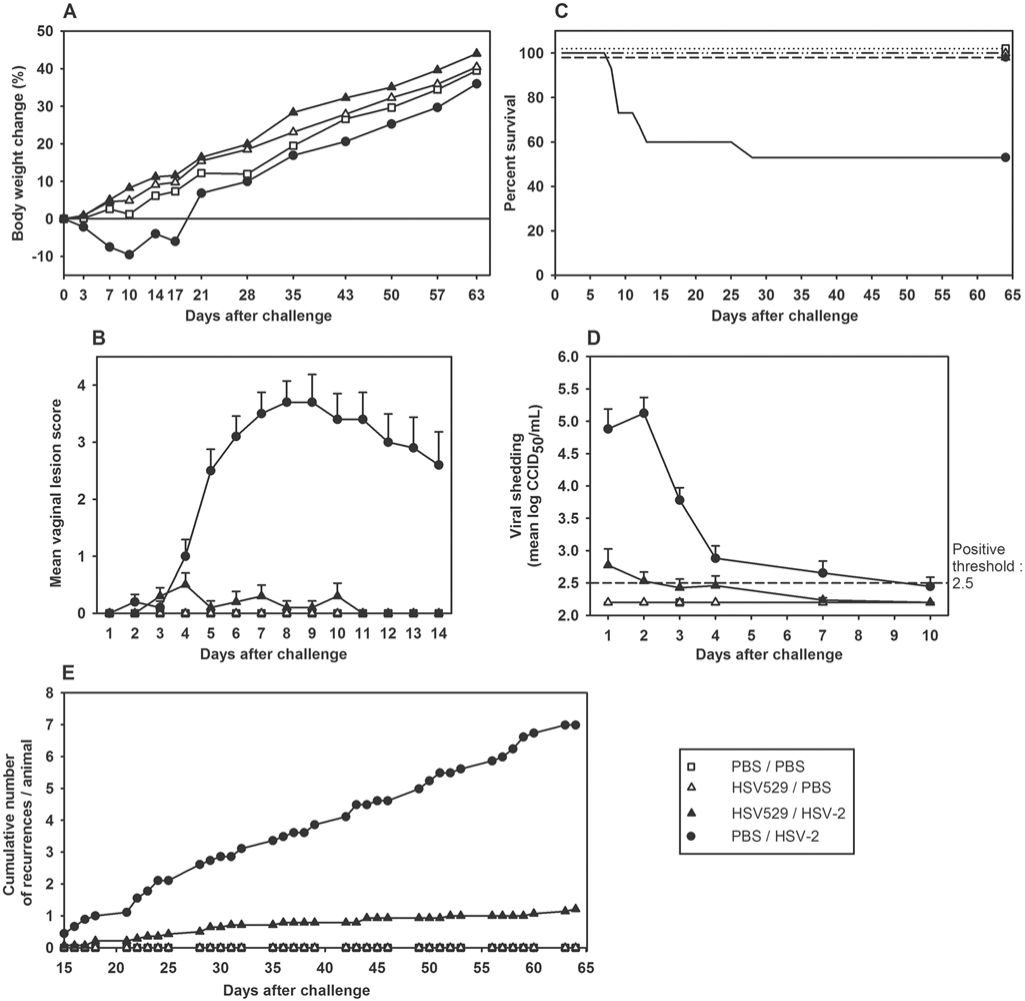
HSV529 immunization protects guinea pigs from the effects of HSV-2 vaginal challenge. Guinea pigs were immunized with HSV529 (10^6^ CCID_50_; n = 30) or PBS (n = 25) by the i.m. route on days 0 and 21. On day 48, 15 animals in each group were challenged with an intravaginal inoculation of HSV-2 (G strain; 10^5^ CCID_50_). The remaining animals received a mock challenge of PBS. (A) Mean body weight change after HSV-2 challenge. (B) Mean vaginal lesion score after HSV-2 challenge. (C) Percent survival after HSV-2 challenge. (D) HSV-2 viral shedding after challenge. (E) Cumulative number of recurrent lesions per animal. *Dead or euthanized animal. Error bars represent standard error of the mean.

Vaginal HSV-2 infection in guinea pigs has many of the same features of the human disease including an acute phase, viral shedding, viral latency, and recurrent lesions. In the guinea pig model the HSV-2 challenge dose is intended to infect 100% of the animals but is not necessarily lethal, although some animals may die from the infection or may need to be euthanized due to severe disease. Guinea pigs immunized with HSV529 (10^6^ CCID_50_, i.m.) 48 and 27 days before HSV-2 vaginal challenge (G strain, 10^5^ CCID_50_) were protected from subsequent weight loss (n = 14; Fig. 4A) and from developing vaginal lesions (n = 14; Fig. 4B). Control animals (n = 15) attained a peak mean acute lesion score of 3.7 ± 1.4 at p.i. day 8, and 7 of the animals died or were euthanized between p.i. days 8 and 28. In contrast, animals immunized with HSV529 had a maximum mean acute lesion score of only 0.5 ± 0.7 on p.i. day 4 and none of the animals died (Fig. 4C). Overall, lesion scores during all the acute phase were significantly lower in the HSV529-vaccinated group than in the control group (p = 0.0003, analysis of variance). Whereas all 15 control animals shed vaginal virus and had a geometric mean titer of 5.1 ± 0.9 log_10_ CCID_50_ on p.i. day 2, only 5 of the 14 immunized animals were shedding virus on p.i. day 2 with a geometric mean titer of 2.5 ± 0.5 log_10_ CCID_50_ (Fig. 4D). Overall, viral titers between p.i. days 2 and 4 were significantly lower in the immunized group than in the control group (all p-values < 0.038, Wilcoxon test).

In the guinea pig model, recurrent lesions begin to appear approximately 2 weeks after challenge as HSV-2 latency is established in the corresponding innervating lumbosacral ganglia. Acute phase lesions (p.i. days 0−14) are still present and can persist for up to 4 weeks after infection. Thus, to assess the extent of recurrent disease in infected guinea pigs, new lesions appearing between p.i. days 15 and 65 were tallied daily for control and immunized animals (Fig. 4E). Control animals steadily accumulated new lesions during the observation period, reaching a mean cumulative total of nearly 7 episodes per animal by p.i. day 65, whereas HSV529-immunized animals experienced a mean total of only 1.2 episodes per animal. Overall, throughout the entire latent phase period, the mean cumulative total of recurrent lesions was significantly lower in the HSV529-vaccinated group than in the control group (p < 0.0001, analysis of covariance). However, similar proportions of animals in each group (7 out of 8 control animals, 88%; and 10 out of 14 HSV529-vaccinated animals, 71%) had at least one recurrent lesion.

At the end of the experiment, the proportion of animals bearing latent HSV-2 DNA in their lumbosacral ganglia was significantly lower in the HSV529-vaccinated group (14.3%) than in the control group (100%; p = 0.0001, Chi-square test) (Table 1). The geometric mean of the latent viral DNA content in the ganglia was also significantly lower in the HSV529-vaccinated group (2.54 ± 0.40 log_10_ copies/animal) than in the control group (4.29 ± 0.47 log_10_ copies/animal; p < 0.0001, analysis of variance).

**Table 1:** Immunization with HSV529 protects naïve and HSV-1 seropositive guinea pigs from latent HSV-2 infection in lumbosacral ganglia.

Model	Treatment	Vaginal challenge	Number of animals with HSV-2 DNA in LSG, n/N (%)	Mean HSV-2 DNA content, log_10_ copies/animal ± sd (range)
**Naïve guinea pigs** ^a^	PBS	PBS	0/4 (0%)	≤ LLOD (2.38)(NA)
	HSV529 (10^6^ CCID_50_)	PBS	1*/15 (7%)	≤ LLOD (2.38)(≤ LLOD−2.86)
	HSV529 (10^6^ CCID_50_)	HSV-2 (G)10^5^ CCID_50_	2/14 (14.3%)*	2.54 ± 0.40(≤ LLOD−3.56)
	PBS	HSV-2 (G)10^5^ CCID_50_	8/8 (100%)	4.29 ± 0.47(3.61−4.93)
**HSV-1-seropositive guinea pigs** ^b^	PBS/PBS	PBS	0/2 (0%)	≤ LLOD(NA)
	PBS/PBS	HSV-2 (G)2 x 10^6^ CCID_50_	3/6 (50%)	3.14 ± 0.91(≤ LLOD−4.56)
	HSV-1 (KOS, 10^6^ CCID_50_) /PBS	HSV-2 (G)2 x 10^6^ CCID_50_	9/13 (69.2%)	3.40 ± 0.77(≤ LLOD−4.44)
	HSV-1 (KOS, 10^6^ CCID_50_) /HSV529 (10^6^ CCID_50_)	HSV-2 (G)2 x 10^6^ CCID_50_	4/15 (26.7%)**	2.56 ± 0.34**(≤ LLOD−3.48)

CCID_50_, cell culture infection dose 50 (1 CCID_50_ of virus infects 50% of 10wells of a 96-well plate); LLOD, lower limit of detection; LSG, lumbosacral ganglia; n, number of animals with HSV-2 DNA > LLOD; N, total number of animals; NA, not applicable; PBS, phosphate-buffered saline; sd, standard deviation.

* Significantly lower than in the unvaccinated control group, p = 0.0001.

** The number of animals with latent HSV-2 DNA (p = 0.024) and the mean HSV-2 DNA content (p = 0.0008) were both significantly lower than the respective value in the unvaccinated control group.

^a^ In the naïve guinea pig model, animals were immunized with PBS or HSV529 i.m. in weeks 0 and 3, challenged with HSV-2 in week 7, and evaluated at week 16 or 17.

^b^ In the HSV-1 seropositive guinea pig model, animals were infected with intranasal HSV-1 in week 0, immunized with PBS or HSV529 i.m. in weeks 7 and 10, challenged with HSV-2 in week 14, and evaluated at week 24.

Two injections of HSV529 vaccine also induced sustained HSV-2-specific neutralizing antibody titers of 3.7 ± 0.3 log_10_ SN_50,_ 9 days after the second immunization. These responses remained stable during the experiment and did not increase after HSV-2 vaginal challenge (data not shown).

### HSV529 immunization also effectively protects HSV-1 seropositive guinea pigs

Because HSV-1 antibodies may affect HSV-2 vaccine efficacy in humans [6] and because HSV-1 seropositivity is the most common human situation, we examined the protective efficacy of the HSV529 immunization in HSV-1-seropositive guinea pigs that had been previously infected at week 0 with HSV-1 (KOS strain; 10^6^ CCID_50_) by the intranasal route [12]. All animals inoculated with HSV-1 were found seropositive at week 4 and were then immunized at weeks 7 and 10 with HSV529 (10^6^ CCID_50_) or PBS. At week 14, all HSV-1 seropositive animals and 14 naïve animals were challenged by the vaginal route with a dose of HSV-2 (G strain, 2 x 10^6^ CCID_50_) that was 20-fold higher than the challenge dose normally used for naïve guinea pig experiments. HSV-1 antibodies reduce symptomatic HSV-2 infection so this higher dose was used to ensure HSV-2 disease in all animals.

**Fig 5:**
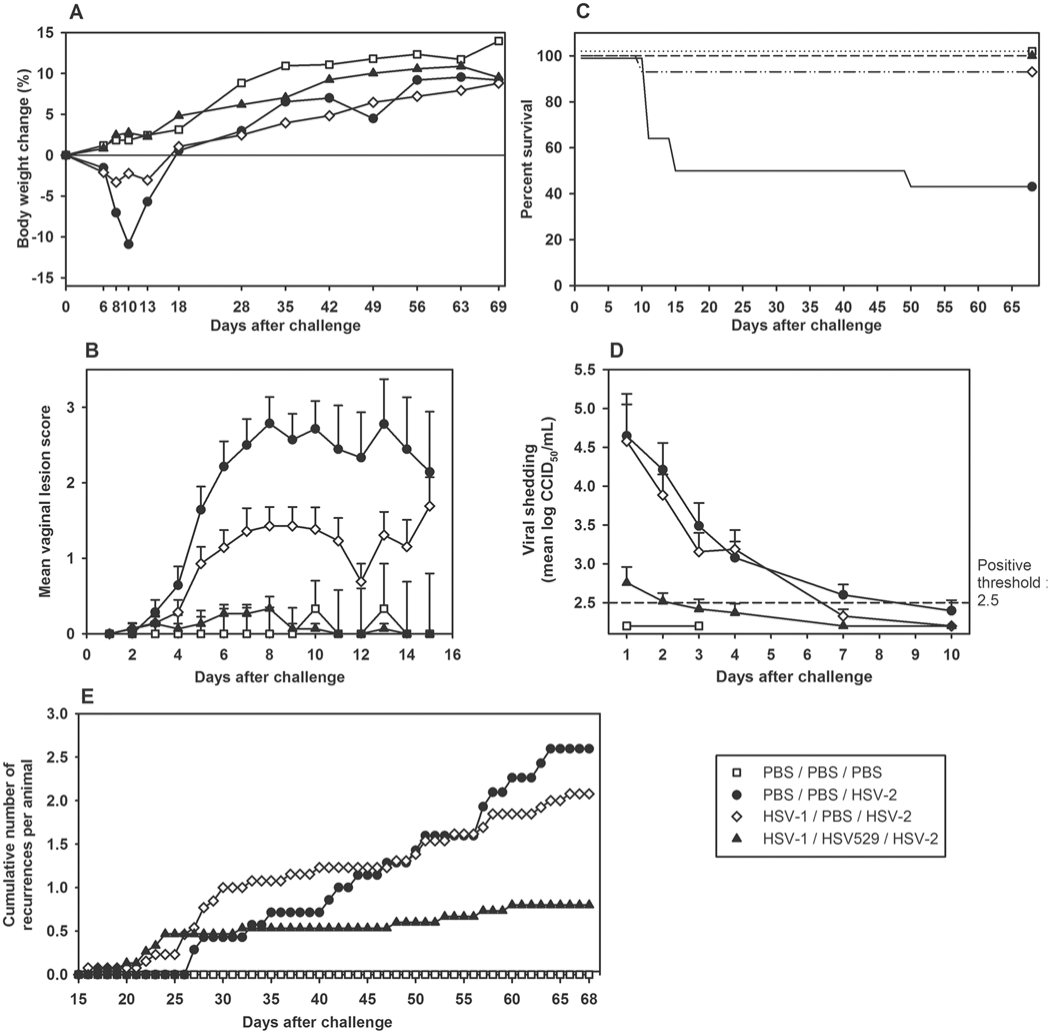
HSV529 immunization protects HSV-1-primed guinea pigs from effects of HSV-2 vaginal challenge. Guinea pigs were inoculated with HSV-1 (KOS strain; 10^6^ CCID_50_; n = 30) or PBS (n = 18) by the intranasal route on day 0. All animal inoculated with HSV-1 were positive for HSV-1 at week 5. At weeks 7 and 10, animals inoculated with HSV-1 were immunized with HSV529 (10^6^ CCID_50_; n = 15) or PBS (n = 14) by the i.m. route. At week 14, all animals except 3 PBS controls were challenged with an intravaginal inoculation of HSV-2 (G strain, 2 x 10^6^ CCID_50_). (A) Mean body weight change after HSV-2 challenge. (B) Mean vaginal lesion score after HSV-2 challenge. (C) Percent survival after HSV-2 challenge. (D) HSV-2 viral shedding after challenge. (E) Cumulative number of recurrent lesions per animal. *Dead or euthanized animal. Error bars represent standard error of the mean.

HSV529 immunization protected most HSV-1-seropositive animals from most HSV-2 genital disease features (Fig. 5). Immunized animals continued to grow and gain weight following HSV-2 challenge; though over time they tended to gain less weight than unchallenged control animals (Fig. 5A). In contrast, HSV-1-naïve control animals lost >10% of their body weight after challenge (p.i. day 10) and HSV-1-primed animals lost 2%. During the 14-day acute phase, genital disease was far less severe in immunized animals than HSV-1 seronegative or HSV-1 seropositive but PBS immunized animals, and none of the HSV529-immunized animals died (Fig. 5B and 5C). In HSV529-immunized animals, 7 (40%) remained lesion-free, 8 had lesion scores no higher than 2, and the mean lesion score was significantly lower than the mean scores in the two other HSV-2-challenged groups regardless of their HSV-1 serological status (all p-values ≤ 0.0002, analysis of variance). In non-vaccinated animals, lesions developed in all 14 HSV-1-naïve control animals, the maximum mean lesion score was 2.8 on p.i. day 8, and 7 of the animals died or were euthanized. Lesions developed in 13 of the 14 HSV-1-seropositive animals, with a maximum mean lesion score of 1.4 on p.i. day 8 and one death.

Vaginal shedding of HSV-2 virus was much lower in HSV529-immunized animals than in either challenged control group, as the mean viral titer was not above the positive threshold on p.i. day 2 and shed virus was not detected in 6 out of 15 (40%) animals (Fig. 5D). In contrast, both control groups were shedding high titers of HSV-2 virus at p.i. day 2 (>3.9 log_10_ CCID_50_/mL), which did not decline to below the positive threshold until at least p.i. day 7. Only 3 out of 14 (21%) HSV-1-naïve control animals and 2 out of 14 (14%) HSV-1-seropositive animals remained free of detectable virus. The mean viral loads in the HSV529-immunized group on p.i. days 2, 3, and 4 were all significantly lower than in the two control groups regardless of HSV-1 serological status (all p-values < 0.0001, analysis of covariance).

HSV529-immunized animals exhibited significantly fewer episodes of recurrent disease than non-immunized animals (p < 0.0001, analysis of variance; Fig. 5E). By p.i. day 68, the final cumulative rates of recurrent disease per animal were 0.8 episodes in the HSV529-immunized group (n = 15), 2.6 episodes in the HSV-1-naïve control group (n = 7), and 2.1 episodes in the HSV-1-seropositive group (n = 13). However, the rates of HSV-1 seropositive animals with at least one recurrent episode in the HSV529-immunized group (9 out of 15 animals, 60%) and in the non-immunized group (11 out of 13 animals, 85%) were not significantly different (p = 0.22, Fisher’s exact test).

Immunization with HSV529 also reduced HSV-2 latency in HSV-1 seropositive animals (Table 1). The frequency of animals in the HSV529-vaccinated group harboring latent HSV-2 DNA in their lumbosacral ganglia after HSV-2 challenge (4 out of 15 animals, 26.7%), as well as the mean HSV-2 DNA content in these ganglia (2.56 ± 0.34 log_10_ copies/animal) were both significantly lower than in the control group (9 out of 13 animals, 69.2%, p = 0.024, Chi-square test; mean content: 3.40 ± 0.77 log_10_ copies, p = 0.0008, analysis of variance).

### Absence of HSV529 replication and/or propagation in sensitive models of HSV-2 pathogenicity

**Fig 6:**
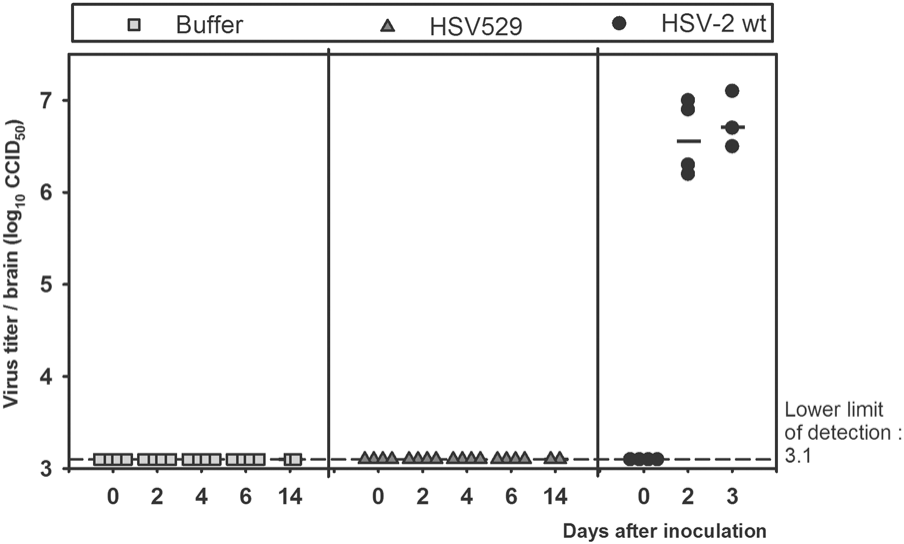
HSV529 does not propagate in the brains of 4−6-day-old suckling mice. Four- to 6-day-old sucking mice received an intracranial injection of vaccine buffer (gray squares), HSV529 (gray triangles, 5 x 10^5^ CCID_50_), or wild-type (wt) HSV-2 186 syn+-1 (black circles, 10 CCID_50_). Brains were collected on p.i. days 0 (4 hours p.i.), 2, 4, 6, and 14, and from animals that died during the experiment. The titer of each animal is represented by an individual symbol and the mean titer is represented by a horizontal bar. Virus titers were determined on AV529-19 cells.

To verify that the HSV529 vaccine candidate is unable to replicate or produce progeny virus and cause disease *in vivo*, we evaluated it in three sensitive models of HSV-2 infection and pathogenicity. Four- to six-day-old suckling mice received an intracranial inoculation of buffer, HSV529 (5 x 10^5^ CCID_50_), or HSV-2 186 syn^+^-1 (10 CCID_50_) (Fig. 6). All mice inoculated with wild-type HSV-2 died by p.i. day 3 and had high intracranial titers (> 6 log_10_ CCID_50_/brain) of virus as soon as D2 after inoculation. In contrast, none of the mice inoculated with HSV529 developed disease or died during the 14 days after injection, despite having received a dose of virus 50,000 times higher than the dose of wild-type HSV-2. No virus was detected (titers under the limit of titration of 3.4 log_10_ CCID_50_/brain) in the brains of these animals collected between p.i. days 0 and 6 (n = 4 per time point) or on p.i. day 14 (n = 1). Residual HSV529 genomic DNA was detected by qPCR in the brains of animals collected 4 hours after injection on day 0 (not shown).

To determine whether HSV529 replicates and propagates in muscle tissue of severely immunocompromised animals, we inoculated SCID/Beige-mice with PBS, HSV529 (4 x 10^6^ CCID_50_), heat-inactivated HSV529 (4 x 10^6^ CCID_50_ equivalents), or HSV-2 186 syn^+^-1 (500 CCID_50_) in the gastrocnemius muscle (Table 2). Whereas 7 out of 8 animals injected with wild-type HSV-2 died within 3 weeks, none of the animals injected with PBS, HSV529, or heat-inactivated HSV529 developed disease or died during the 28 days after injection (Table 2). In animals inoculated with wild-type HSV-2, virus was not detected in muscles collected on the day of injection (n = 4) but was detected in all muscles collected after death or on p.i. day 28. In animals inoculated with HSV529, residual virus from the i.m. injection was detected in muscles collected on the day of injection (n = 4) if extracts were plated on AV529-19 cells, but no viruses were detected in any muscles collected on p.i. day 28 (n = 8) if extracts were plated on either AV529-19 or Vero cells. No virus was detected in the muscles of any animals inoculated with heat-inactivated HSV529 (n = 12).

**Table 2:** Lack of HSV529 propagation in sensitive models of HSV-2 replication.

Model	Treatment	Dose	Evaluation time points	Number of animals (N)	Survival, n/N (day)	Virus detected on AV529 cells, n/N (day)	Virus detected on Vero cells, n/N (day)
**Brain of suckling mouse**	Buffer	20 μL	D0, 2, 4, 6, 14	-20 (4/group)	NA*	0/18 (all days)	
	HSV529	5 x 10^5^ CCID_50_	D0, 2, 4, 6, 14**	17 (4/group)	NA*	0/17 (all days)	
	wt HSV-2	10 CCID_50_	D0	11 (4/group)	NA*	0/4 (D0)	
			D2			4/4 (D2)	
			D3			3/3 (D3)	
**Gastrocnemius muscle of SCID/Beige mouse**	PBS	20 μL	D28	4	4	0/3	0/1
	heat-inactivated HSV529	4 x 10^6^ CCID_50_ eqs.	D0	4	NA	0/4	0/1
			D28	12	12/12	0/12	0/1
	HSV529	4 x 10^6^ CCID_50_	D0	4	NA	4/4	0/4
			D28	12	12/12	0/12	ND
	wt HSV-2	500 CCID_50_	D0	4	NA	0/4	ND
			D8−D28	8	1/8 (D28)	7/8	8/8
**Guinea pig vaginal mucosa**	PBS	100 μL	D3, 5, 7, 10, 13, 31	4	4/4	0/4 (all days)	0/4 (all days)
	heat-inactivated HSV529	2.0 x 10^7^ CCID_50_ eqs.	D3, 5, 7, 10, 13, 31	8	8/8	0/8 (all days)	0/8 (all days)
	HSV529	2.0 x 10^7^ CCID_50_	D3, 5, 7, 10, 13, 31	8	8/8	0/8 (all days)	0/8 (all days)
	wt HSV-2	6700 CCID_50_	D3	8	8/8	4/8	6/8
			D5		8/8	4/8	4/7
			D7		8/8	1/8	1/7
			D10		6/8	0/6	0/6
			D13		5/8	0/5	0/5
			D31		5/8	0/5	0/5

CCID_50_, cell culture infection dose 50 (1 CCID_50_ of virus infects 50% of the cells in the well of a 96-well plate); CCID_50_ eqs., CCID_50_ equivalents (the CCID_50_ in the sample prior to heat inactivation); D, day; n, number of animals exhibiting the characteristic; N, total number of animals assessed; NA, not applicable (gastrocnemius muscles were collected from D0 animals 4 hours after inoculation; ND, not determined; PBS, phosphate-buffered saline; SCID, severe-combined immunodeficiency; wt, wild-type.

*Most animals in these groups were euthanized on specific days to assess cranial viral titers. In the HSV529 group, 3 mice died 4 hours after inoculation, probably due to the injection procedure. In the wt HSV-2 group, all remaining animals in the third group were dead on day 3.

** Only one mouse was tested at the D14.

To determine if HSV529 replicates and is pathogenic in genital mucosa, we inoculated guinea pigs by the vaginal route with PBS, heat-inactivated HSV529 (2.0 x 10^7^ CCID_50_ equivalents), HSV529 (2.0 x 10^7^ CCID_50_), or an approximate LD_50_ of HSV-2 186 syn^+^-1 (6700 CCID_50_) and monitored them between p.i. days 0 and 31 for clinical signs of genital herpes and viral shedding (Table 2). As expected, all animals inoculated with wild-type HSV-2 developed acute genital herpes; 3 of them died or were euthanized by p.i. day 10; and 6 out of 8 animals shed virus detectable on AV529-19 or Vero cell cultures. Animals inoculated with HSV529 or heat-inactivated HSV529 displayed no clinical signs of disease and shed no virus detectable on AV529-19 or Vero cell cultures.

## Discussion

Investigative preparations of the replication-defective *dl5-29* virus, the earlier versions of the vaccine candidate, do not cause disease in sensitive animal models and are known to be immunogenic and effective at preventing HSV-2 infection and disease in animal models of genital herpes [8–14]. However, it was necessary to confirm these activities and the non-replicative status of the HSV529 clinical preparation prior to its evaluation in clinical trials. We demonstrated that prophylactic vaccination with the HSV529 vaccine was immunogenic and protective in these well-known animal models of genital herpes, including HSV-1-seropositive guinea pigs. HSV529 was also unable to replicate (under the method thresholds) or cause disease *in vivo* in two very sensitive mouse models of HSV replication and in guinea pig vaginal mucosa.

As previously shown in mice, we also found that in guinea pigs HSV529 was significantly more immunogenic if it was delivered by the i.m. route rather than by the s.c. route. This may be an important feature of HSV529 immunization, as i.m. will be the clinical route of administration. HSV529 vaccination in mice induced humoral and cellular immune responses, including production of HSV-2-neutralizing antibodies. The humoral responses were all enhanced following the booster vaccination.

The humoral response in mice included a Th1/Th2 characteristic response reflected by production of IgG subtypes 1 and 2a. Our findings complement those of other studies reporting induction of HSV-2-neutralizing antibodies in guinea pigs and mice and induction in mice of CD8+/IFNγ+ splenic lymphocytes following vaccination with *dl5-29* [8, 13] and of CD4+/IFNγ+ splenic lymphocytes following vaccination with ACAM529 [11]. Although we did not analyze T-cell subsets, the cellular responses we observed included IFNγ+ splenocytes, which is consistent with CD4+ helper cells having a Th-1 phenotype. However, in contrast to previous findings [8, 12], the representation of CD8+ cells in this population was probably low because we stimulated the splenocytes with heat-inactivated virus rather than live HSV-2-infected cells. Production of IL-5+ cells is consistent with a CD4+/Th2 phenotype. Taken together, these results suggest that vaccination with HSV529 results in a comprehensive and Th1/Th2 characteristic immune response with sufficient immunological memory to allow the humoral arm of the response to be boosted by a second vaccination.

The strong immunogenic profile of HSV529 is consistent with its highly effective protection against vaginal HSV-2 challenge in animal models of genital herpes. As previously reported for *dl5-29* and ACAM529, HSV529 vaccination largely or completely prevented virtually all consequences of vaginal HSV-2 infection in naïve mice and guinea pigs, including weight loss, acute disease, lethality, viral shedding, recurrent disease, and viral latency (under the method thresholds). In mice, HSV529 vaccination was 90% effective against lethality whereas vaccination with *dl5-29* or ACAM529 was 100% effective in three previous studies [8, 11, 13] and 75% effective in a fourth study [9]. However, in our study, the one animal that died in the HSV529-vaccinated group had no signs of disease and did not succumb until p.i. day 24 so this animal may have died from a cause other than HSV-2 infection. Thus, these results confirm the efficacy of the highly purified HSV529 vaccine candidate.

Because a large proportion of the global population is HSV-1 seropositive and this has been found to be an important factor in the efficacy of HSV-2 vaccines in clinical trials [6], developing a vaccine for these individuals represents an additional challenge. HSV529 may be an effective vaccine in HSV-1-seropositive individuals because *dl5-29* was shown to be similarly immunogenic and protective in naïve as well as HSV-1-seropositive guinea pigs [12]. In both of these animal models, vaccination with *dl5-29* generated similar titers of HSV-2-neutralizing antibodies and elicited comparable reductions in peak mean acute lesion scores, vaginal viral shedding, cumulative totals of recurrent lesions, and latent viral DNA loads despite the use of a vaginal HSV-2 challenge dose that was 20-fold higher in HSV-1-seropositive animals. As expected, HSV529 was similarly effective in naïve and HSV-1-seropositive guinea pigs, as vaccination reduced acute and recurrent lesions, viral shedding (compare Figs. 4 and 5), and viral DNA loads in ganglia (Table 1), even with the higher HSV-2 challenge dose required to infect HSV-1-seropositive animals.

In the different protection studies, it should be noted that the threshold of titration was of 2.5 log_10_ CCID50 and a possibility still remained that wild type HSV-2 was shed in the genital secretions of challenged mice and guinea pigs at a level below the positive threshold value (or LLOD) that was set.

It is interesting to note that the recurrent disease per animal measured by 65 days after challenge was found substantially higher in the first experiment guinea pig protection study than in the second one even though the challenge dose was 10-fold higher. The frequencies were of 7.0 and 2.6 recurrences/animal for the PBS/HSV-2 and PBS/PBS/HSV-2 groups, respectively, reflecting the known variability of susceptibility to infection from one experiment to another.

HSV529 lacks two genes essential for viral DNA replication and its *dl5-29* predecessor is absolutely replication-defective in normal cells *in vitro* and in mouse nasal mucosa *in vivo* [10, 14]. In previous *in vivo* studies, 10^6^ pfu of the *dl5-29* virus was also unable to cause disease or lethality after intracranial administration to 4-week-old BALB/c mice or after i.m. administration to severely immunocompromised SCID mice [13]. Both of these models have been also used to assess the safety of other HSV vaccine candidates [15]. Further, Awasthi S *et al*. showed that the LD_50_ values of wild-type HSV-2 delivered by the intracerebral route to 20-day-old BALB/c mice (< 5 pfu) and by the intramuscular route to adult SCID mice (5 x 10^4^ pfu) were much lower than the LD_50_ of i.m. HSV-2 in adult BALB/c (1.6 x 10^6^ pfu). We confirmed and extended the earlier *dl5-29* findings in two models of HSV-2 replication that are arguably more sensitive than the previous models used by Hoshino et al. [13] and we also measured the titers of virus in the animals following inoculation with wild-type HSV-2 or HSV529. First, compared to an intracranial dose of wild-type HSV-2 (500 CCID_50_) that killed all 4−6-day-old sucking mice within 3 days and produced high intracranial titers of virus, a 50,000-fold higher dose of HSV529 produced no disease or detectable virus in the brains of the mice (under the method threshold) at any time point. Second, no replication or disease occurred after i.m. administration of 4 x 10^6^ CCID_50_ of HSV529 to severely immunocompromised SCID/Beige mice, which in addition to the B and T cell deficiencies of the SCID background also lack natural killer cells. Finally, as shown previously for *dl5-29* (10^6^ pfu) [8], no disease or viral shedding (under the method threshold) occurred after vaginal inoculation of guinea pigs with a much higher dose of HSV529 (2.2 x 10^7^ CCID_50_). These results confirm the non-virulent and replication-defective phenotypes of HSV529 and underscore the considerable attenuation margin of HSV529.

Belshe et al. in a phase III clinical trial showed that the gD2 vaccine containing the adjuvants alum and monophosphoryl lipid A was efficacious against HSV-1 infection but failed to induce significant protection against primary HSV-2 disease despite a good efficacy profile in preclinical studies [7]. Although the HSV529 vaccine candidate has been evaluated by the i.m. route for protection against HSV-2 (G strain) infection in animal models, it has never been compared side-by-side to the gD2 in alum-monophosphoryl lipid A vaccine candidate.

However, Hoshino et al. compared the protection induced by the HSV529-predecessor vaccine, *dl5-29*, given by the s.c. route to the gD2-adjuvanted vaccine given by the i.m. route in HSV-1-positive or-negative guinea pigs [12], and Delagrave et al. compared the protection induced by ACAM529 in mice to that of gD2 adjuvanted with CpG and alum, both given by the i.m. route [11]. Both studies showed that either *dl5-29* or ACAM529 afforded better protection than the adjuvanted-gD2 vaccine against infection with HSV-2 strain 333, with significantly fewer disease symptoms and lower levels of latent dorsal root ganglia infection. Those data, as well as the capabilities of the HSV529 vaccine to present a greater number and variety of epitopes and to exploit different antigen presentation mechanisms, suggest that HSV529 could afford better protection in humans than the adjuvanted-gD2 vaccine.

In this comprehensive investigation of HSV529 prior to its evaluation in clinical trials, we confirmed that it is immunogenic and provides high level of protection against HSV-2 infection and disease regardless of HSV-1 serostatus at the time of vaccination. Importantly, it was also unable to replicate in two very sensitive mouse models of viral replication, as well as in guinea pig vaginal mucosa. These results suggest that HSV529 is appropriate for clinical investigation, that it is likely to be as effective in subjects who are also HSV-1 seropositive.
